# “*I was Treated by the Program, the Therapist, and Myself*”: Feasibility of an Internet-Based Treatment Program for Gambling Disorder

**DOI:** 10.1007/s10899-023-10199-x

**Published:** 2023-03-31

**Authors:** Anna Westh Stenbro, Stine Moldt, Jakob Winther Eriksen, Lisbeth Frostholm

**Affiliations:** 1 Research Clinic for Gambling Disorder, Aarhus University Hospital, Universitetsbyen 4, 1st Floor, 8000 Aarhus C, Denmark; 2 Research Clinic for Functional Disorders and Psychosomatics, Aarhus University Hospital, Aarhus, Denmark; 3 Department of Clinical Medicine, Aarhus University, Aarhus, Denmark

**Keywords:** Gambling disorder, Internet-based treatment, Cognitive behavioral therapy, Therapist-guided, Feasibility, Acceptability

## Abstract

The majority of people with problematic gambling behavior never seek or receive professional help. Internet-based treatment approaches have been shown to help patients overcome practical and psychological barriers associated with face-to-face therapy. In this uncontrolled pilot study, we explored the feasibility of the eight-module therapist-guided internet-based treatment program *SpilleFri* (“Free from Gambling”) for patients with gambling disorder (GD). We included 24 patients seeking treatment at a Danish hospital-based treatment clinic. The focus of the feasibility study was evaluation of recruitment and retention rates, data completion rates, treatment response, satisfaction, and program utility. In addition, a series of semi-structured interviews were conducted to explore patient-perceived acceptability and potential barriers to treatment completion and positive outcome. Treatment acceptability among therapists was examined in a focus group interview. Sixteen of the included patients completed the program, yielding an acceptable treatment dropout rate (29.17%), and 82.35% of treatment completers provided full data at all assessment points. Overall, patients were satisfied with the treatment received, and patient interviews highlighted multiple psychological as well as practical benefits associated with the treatment form and content. Patients with more severe gambling symptoms at baseline might be more likely to drop out before treatment completion than patients with less severe symptoms. The results suggest that *SpilleFri* may be a feasible alternative to face-to-face treatment for GD. However, the uncontrolled design and small sample size of the study limit the robustness of the findings. In the future, the effect of *SpilleFri* treatment should be examined in a randomized controlled trial.

*Clinical trial registrations*: The study protocol was prospectively registered at clinicaltrials.gov (TRN: NCT05051085; registration date: September 21, 2021).

**Supplementary Information:**

The online version contains supplementary material available at 10.1007/s10899-023-10199-x.

## Introduction

Gambling disorder (GD) is characterized by persistent and recurrent maladaptive patterns of gambling behaviors. In ICD-10, GD is defined as an impulse disorder with a pattern of continued gambling despite negative physical, psychological, and social consequences (World Health Organization, 1993). According to the Diagnostic and Statistical Manual of Mental Disorder, 5th edition (DSM-5), GD is diagnosed when at least four of nine symptoms are present during a 12-month period (e.g., development of tolerance, loss of control, gambling to compensate for losses) (American Psychiatric Association, 2013). Internationally, the lifetime prevalence of GD is estimated to be around 0.4–1.0% in the general adult population (American Psychiatric Association, 2013). In Denmark, a prevalence estimate of 0.67% of the general adult population scoring above the cut-off for serious gambling problems in the past year, measured via self-report on the Problem Gambling Severity Index (*PGSI)*, was found in a general population study from 2022 (Rambøll, 2022). People with GD often suffer from comorbid psychiatric conditions such as depression (with estimated comorbidity rates of 29.2%), alcohol abuse (21.2%), anxiety disorders (17.6%), and other forms of addictive disorders (7%) (Dowling et al., 2015). Without treatment, patients risk serious long-term consequences of GD, including severe psychosocial and financial problems (Cowlishaw et al., 2016; Gainsbury et al., 2014).

Fortunately, the evidence base for psychological treatment options for GD is growing (Ribeiro et al., 2021). In a recent systematic review of randomized controlled trials, Ribeiro and colleagues found the effectiveness of cognitive behavioral therapy (CBT) to be established in seven randomized controlled trials (Ribeiro et al., 2021). Treatment programs utilizing motivational interviewing (MI) or mindfulness interventions also show promising results (Sagoe et al., 2021).

Despite efforts to make effective treatment options widely accessible, remarkably few individuals with GD seek and receive treatment. Studies have found that less than 10% of persons with GD ever seek help (Statens Folkhälsoinstitut, 2010; Suurvali et al., 2009). Paired with generally high attrition rates reported in treatment studies (averaging 42%), the actual number of patients receiving effective treatment dosages is alarmingly low (Dunn et al., 2011; Melville et al., 2007; Westphal, 2007). Studies of barriers to treatment in patients with GD indicate that they might be reluctant to seek treatment for both practical and psychological reasons (Suurvali et al., 2009). Practically, patients might consider attending treatment too costly, time-consuming, and/or inflexible to fit into their everyday lives (Hodgins & El-Guebaly, 2000; Hodgins et al., 2009; Pulford et al., 2008; Rockloff & Schofield, 2004). Psychological barriers to treatment seeking include a desire in gamblers to handle their problems on their own, unwillingness to admit the full extent of their gambling problems as well as shame, secrecy, embarrassment, pride, and fear of stigma (Suurvali et al., 2009).

Internet-based treatment includes different types of remotely delivered psychological treatment programs and has been put forward as a way of mitigating multiple of these barriers (van der Maas et al., 2019) while at the same time facilitating treatment accessibility and broadening availability. Compared to face-to-face treatment, internet-based treatment is unrestrained by geographical distance and interferes less with work and family obligations, thus reducing strain on daily activities. In addition, the discretion of “*logging into treatment*” or receiving psychological help by phone as compared to showing up at a treatment facility seems to be less stigmatizing for patients (Marques et al., 2010).

To date, research on the acceptability and effect of internet-based psychological treatment for GD is limited. In a recent meta-analysis, Sagoe and colleagues found large effects of internet-based cognitive behavioral therapy (iCBT) for gambling symptoms as well as small significant effects on gambling frequency and money lost (Sagoe et al., 2021). The treatment programs included in the meta-analysis varied greatly by content and context: For example, eight out of 13 studies evaluated unguided treatment programs (e.g., online support forums), and the intervention length varied between single sessions and up to 28 sessions. Moreover, none of the studies evaluated treatment effects in clinical populations receiving a GD diagnosis after clinical assessment, and none of the studies evaluated internet-based treatment programs embedded within existing addiction treatment services. Guided treatment programs were found to be more effective than non-guided treatment. Contrary to expectation, the meta-analysis found more severe gambling symptoms at baseline to be associated with *better* gambling symptom outcome after internet-based treatment.

The aim of the present study was to evaluate the feasibility of a proposed model for internet-based treatment for GD as well as its acceptability among users and therapists delivering the intervention. A treatment program for GD, called *SpilleFri* (Danish for “Free from Gambling”) was developed and implemented within an existing Danish gambling disorder treatment clinic. Specifically, we aimed to evaluate (a) the dropout rate to assess acceptability, (b) data completeness rate to assess feasibility of the study protocol, (c) changes in gambling behavior, GD symptoms, and secondary outcome variables pre- and post-intervention to determine potential benefits and harms of the intervention, (d) the extent of use of the treatment platform, (e) the utility and experiences of the intervention from the patient’s perspective, and (f) the utility and experiences of delivering the intervention from the therapists’ perspective. Apart from the dropout rate and data completeness rate, the evaluations did not include predefined outcome targets, as measurement of these parameters was conducted as proof of concept before undertaking a randomized controlled trial.

## Methods

### Study Design and Setting

This feasibility study took place from September 2021 to April 2022 at the Research Clinic for Gambling Disorder (RCGD), Aarhus University Hospital, Denmark. The study was conducted using a mixed-methods design to enhance the feasibility assessment of the intervention and the procedures (Hansen & Tjørnhøj-Thomsen, 2015). Quantitative methods included descriptive analysis of dropout rates and data completion, changes in GD severity, gambling behaviors, and secondary outcomes post treatment, and the extent of use of the treatment platform. A qualitative sub-study consisting of semi-structured interviews were conducted shortly after treatment initiation (i.e., before the completion of module 4) to explore motivation for change, expectations, and initial experience of the program and after end-of-treatment to explore overall experience of and satisfaction with treatment as well as self-perceived change in symptoms. Furthermore, a focus group interview was conducted with all therapists delivering the intervention to explore therapist intervention acceptability. All participants provided written informed consent, and the study was approved by the Danish Data Protection Agency, Central Denmark Region (ID no. 1-16-02-305-21). The study was pre-registered at clinicaltrials.gov (NCT ID: NCT05051085).

### Participant Recruitment and Eligibility

Patients were recruited and screened for eligibility at the RCGD between September 2021 and January 2022. All patients self-referring for treatment at the RCGD in the recruitment period were screened for eligibility. After initial screening, eligible patients completed a baseline questionnaire, consented to study participation, and underwent diagnostic assessment by experienced clinical psychologists using the DSM-IV-based *Structured Clinical Interview for Pathological Gambling* (*SCI-PG*), modified to fit DSM-5 criteria (American Psychiatric Association, 2013; Grant et al., 2004). To be included in the study, patients had to be between 18 and 60 years of age; meet diagnostic criteria for GD (DSM-5); be able to read, write, and speak Danish to a degree that would allow them to interact with the internet treatment program; have IT skills and access to the internet and a computer and/or tablet; and be willing to participate in an internet-delivered psychological treatment program. Patients were excluded if they had current moderate or severe psychiatric disorder demanding special, individualized treatment (e.g., treatment-demanding depression, personality disorder, and psychotic symptoms). Patients with untreated ADHD, ADD, and cognitive deficits, which would most likely inhibit them from being able to read, understand, and interact with the internet-delivered treatment program, were also excluded.

At the beginning of treatment, patients were asked by their therapist if they would be interested in participating in one or two research interviews regarding their experience of the *SpilleFri* treatment. As most patients were willing to participate in interviews, the recruitment was conducted strategically to include a range of different patients in terms of age, gender, and severity of gambling symptoms, and comorbidity.

### Intervention

The therapist-assisted internet-based treatment program *SpilleFri* was developed at the RCGD by clinical psychologists specialized in the treatment of GD under supervision of a senior researcher with expertise in the development of internet-based interventions. The intervention was developed using a participatory design to ensure that the target patient group experienced the intervention content as relevant and useful. The content was developed de novo, based on a cognitive behavioral treatment program for patients with severe GD used for many years at the RCGD. Selected elements from other therapeutic paradigms such as Motivational Interviewing and Acceptance and Commitment Therapy were included in the treatment content. The technical platform was based on a platform utilized in an empirically tested internet-based treatment program for health anxiety (Hoffmann et al., 2018).

**Fig. 1:**
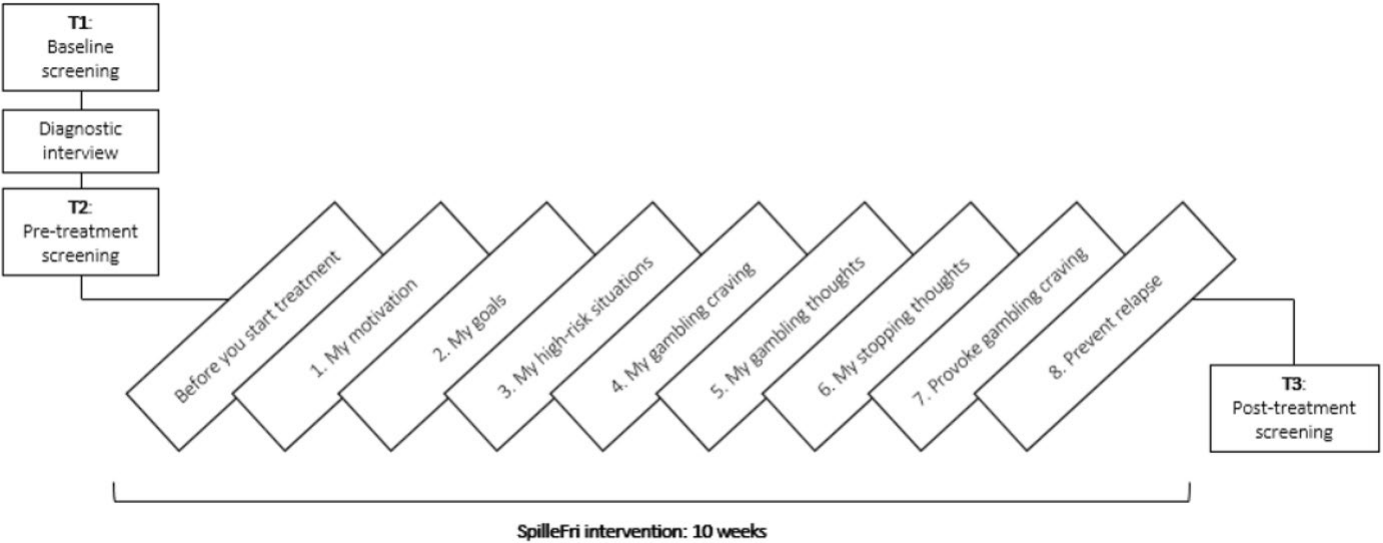
Overview of assessment points and intervention

The resulting 10-week intervention consists of eight modules and one introduction module, covering topics such as cognitive restructuring, coping with craving, exposure, and response prevention (Fig. 1; Table 1). All modules (apart from the introduction module) contain written assignments and multiple choice lists where patients are encouraged to describe and work with thoughts, feelings, and behaviors. The assignments are combined with psychoeducation delivered in texts, illustrations, and videos with GD experts and previous GD patients, and multiple modules have audio clips with mindfulness and visualization exercises. Patients can access the treatment platform at any time of the day using a smartphone, tablet, or computer by a GDPR compliant website, using their NemID (a Danish national two-factor authentication solution).

**Table 1:** Content of treatment modules in *SpilleFri*

Module	Name	Aim	Therapeutic components
Intro	Before you start treatment	Introduction to the treatment and information about gambling disorder	Psychoeducation
1	My motivation	To make patients aware of their motivation to stop gambling and uncover ambivalence	Psychoeducation, motivation
2	My goals	To help patients set goals for gambling and to shift focus from gambling to important life values	Psychoeducation, goals and life values, mindfulness exercises
3	My high-risk situations	To help patients understand which situations are associated with an increased risk of gambling	Registration, situational analysis, coping strategies
4	My gambling craving	To give patients a better understanding of gambling craving and which strategies can be used to cope with craving	Distraction, visualization, mindfulness exercises, relaxation exercises
5	My gambling thoughts	To help patients identify permissive beliefs associated with gambling	Registration, cognitive distortions, psychoeducation
6	My stopping thoughts	To support patients in challenging permissive gambling beliefs and replacing them with stopping thoughts	Registration, challenging beliefs, cognitive restructuring
7	Provoke gambling craving	To support patients in provoking gambling craving and learning to overcome it	Exposure and response prevention, building confidence
8	Prevent relapse	To help patients identify early signs of relapse and develop strategies to cope with them	Psychoeducation, cognitive restructuring, building confidence

The intervention was delivered by experienced clinical psychologists at the RCGD. All psychologists at the clinic have received training and gained some experience using the *SpilleFri* platform in the treatment of GD patients prior to the present study. To ensure fidelity to the treatment concept and aid adherence to study protocols, a treatment manual was provided to therapists at the beginning of the recruitment period.

Patients had a personal therapist assigned who supported them throughout the intervention, guiding them through the modules on a weekly basis by asynchronous written messages via the platform. The assigned therapist was always the psychologist who had conducted the diagnostic assessment, and as such, all patients had met their assigned therapist before treatment onset. Patients could use the written message function whenever they wanted, and the therapist would respond within two workdays. Furthermore, patients had up to four supportive sessions with the therapist during the intervention, including a start-up session. The supportive sessions were hosted in-clinic or via an online video link and were assigned on request of patients or when therapists deemed them necessary to meet individual needs, help patients through particularly challenging treatment phases and/or to avoid dropouts.

### Quantitative Outcomes and Feasibility Criteria

Patient-reported outcomes were collected and managed electronically using the *Research Electronic Data Capture* (REDCap) system for data management (Allen, 2020). Patient-reported data were collected at baseline prior to the diagnostic assessment (T1), before treatment (T2), and at post-treatment (T3) (Fig. 1). Assessment of all primary and secondary outcome variables was conducted at T1 and T3, whereas the T2 assessment only included current gambling behavior and patients' treatment expectations. Clinician-rated outcome measures were collected at T3. Data on patient activity on the treatment platform and contact with therapists were collected during treatment via clinician reports and logged data from the *SpilleFri* platform. Demographic data were collected in the baseline questionnaire, and clinical data were collected through clinic records.

The primary outcome measure was treatment completion rate where a rate above 60% was considered a success. Treatment completion was defined a priori as six or more modules completed (including the introduction module). Lastly, an important feasibility outcome was data completeness, for which the a priori defined feasibility criterion was 70% of treatment completers providing data for the primary gambling outcome variable at T3.

The primary gambling outcome variable was gambling problems measured using the *National Opinion Research Center Screen for Gambling Problems* (NODS) (Wickwire et al., 2008). The NODS is a 17-item self-report questionnaire with a maximum score of 10. On the NODS, 0 points indicate no problematic gambling, 1–2 points mild but subclinical risk for gambling problems, 3–4 points moderate but subclinical gambling problems, and 5–10 points indicate probable GD corresponding to the diagnostic definition of GD in the Diagnostic and Statistical Manual of Mental Disorders, 4th Edition (DSM-IV) (Hodgins, 2004). For the purpose of this study, the NODS was modified to assess past month instead of past year. Gambling outcomes also included self-reported current gambling behavior in the past week, including: The number of gambling sessions, money lost gambling, and time spent gambling at all measurement points. The money lost gambling variable took into account the total sum of money lost minus total winnings.

Symptoms of anxiety and depression were measured on subscales of the 92-item Danish version of the *Symptom Checklist* (SCL-92) (Christensen & Fink, 2010; Derogatis, 1992), namely SCL-dep (6 items, scale range: 0–24) and SCL-anx (4 items, scale range: 0–16) All items are rated on 5point scales ranging from 0 "*not at all bothersome"* to 4 "*very bothersome"*.

Psychological well-being was measured on the *Five Well-Being Index* (WHO-5), a self-reported 5-item scale with a minimum score of 0 (indicating worst imaginable well-being) and a maximum score of 100. (Topp et al., 2015).

Patient quality of life was measured via self-report on a 1 item 11-point scale, where 0 indicates "*the worst possible life”* and 10 indicates "*the best possible life*". The scale is adapted from the Danish questionnaire "*Ungdomsprofilen*" from the University of Southern Denmark. (Bendtsen et al., 2015).

Overall health improvement was measured using the *Clinical Global Impression – Improvement scale* (CGI-I). The CGI was measured by self-report and by clinician rating and consists of one item assessing overall improvement or worsening on a 5-point scale with a minimum score of 1 and a maximum score of 5. (Guy, 1976).

Treatment expectancy and satisfaction was measured using the *Credibility/Expectancy Questionnaire* (CEQ). The CEQ measures self-reported patient treatment expectancy/satisfaction on 6 items rated on a scale of 1–9 or a 0–100%, depending on the item. The scale yields a mean expectancy/satisfaction score, ranging from a minimum of 0% to a maximum of 100%. (Devilly & Borkovec, 2000).

Measurements also included logged data from the *SpilleFri* internet treatment program. Contact with the therapist per patient and patient activity in the treatment program (duration, login time, and frequency) were collected both through therapist report in web-based questionnaire and logged data from the treatment program.

### Statistics

Data were summarized using means and standard deviations. The mean differences from baseline to post-treatment and from T2 to post-treatment were analyzed using paired *t-*tests. Assumptions of normality were assessed both visually using QQ-plots and tested using Shapiro-Wilks test (*n* = 24). Significance was evaluated at alpha = 0.05, and no correction was made for multiple testing. Adherence was measured as the mean number of modules completed. Results are presented using descriptive statistics and reported with frequencies, percentages, means, range, and standard deviation where appropriate.

### Qualitative Data Collection and Analyses

Qualitative data were collected through open-ended questionnaire items in the questionnaire administered to all patients at T3 and through semi-structured interviews with patients included in the qualitative sub-study. Open-ended questionnaire items concerning satisfaction with treatment allowed patients to freely express their experience with treatment, prompting patients to describe both things they liked and disliked about the treatment. The answers to these items were thematically analyzed across patients as recurrent themes were identified. Semi-structured interviews were conducted twice per patient included in the qualitative sub-study: Early in the treatment process (before module 4) to explore motivation for change, expectations, and initial experience of the program and after end-of-treatment to explore overall experience of and satisfaction with treatment as well as self-perceived change in symptoms. All interviews were conducted using an interview guide developed to cover themes relevant to the research objectives. All patients were also allowed to talk freely about the program if they wished to do so. Interviews were recorded, transcribed, and thematically analyzed via coding of relevant themes using the NVivo software (QSR International, 2020). Recurrent themes were identified and described using example statements. Similarly, recorded audio from a focus group with all *SpilleFri* therapists present were transcribed and analyzed thematically. In the focus group, the therapists discussed their experiences of supporting patients through the intervention under the headline: “What does it mean to be a therapist in the context of the *SpilleFri* treatment?”.

## Results

### Participants, Dropout Rate, and Data Completeness

**Fig. 2:**
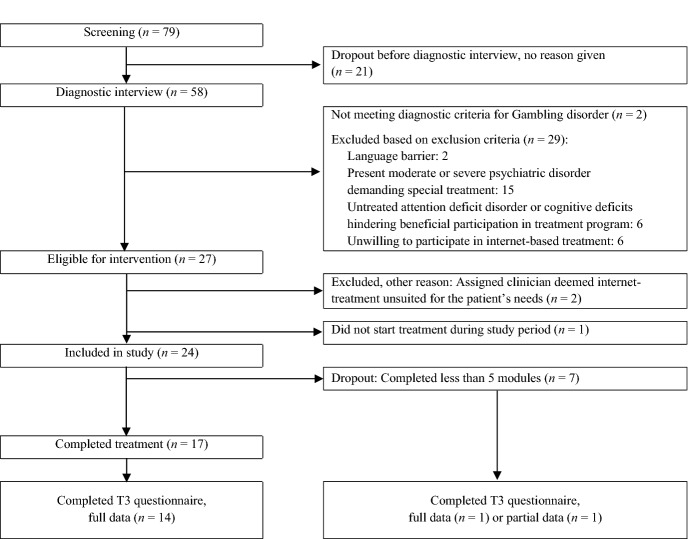
Participant flow

Among 79 patients screened for inclusion, a total of 24 participants were included in the study. Participant flow is described in Fig. 2*.* Of the included participants, seven dropped out before completing the five modules required to be considered ‘completers’ of the intervention corresponding to a dropout rate of 29.17%, which was within the desired rate of < 40%. Of included participants, 16 (66.67%) provided full data on primary outcome measures. Of these, 14 were completers. In total, 82.35% of treatment completers provided full data on primary outcome measures, reaching predefined feasibility criteria of > 60%, and 15 participants provided full data on all outcome measures, including secondary outcomes and treatment satisfaction measures.

Visual inspection of baseline data compared to consecutive dropouts found that patients completing treatment had lower GD severity at baseline (*NODS*, mean = 5.38, SD = 2.18) than patients dropping out of treatment (*NODS*, mean = 7.25, SD = 2.49). Thus, patients with more severe gambling symptoms at baseline appeared more likely to drop out before treatment completion than patients with less severe symptoms (Table 2).

**Table 2:** Baseline data for included patients

	Included (*n* = 24)
Age, mean (SD)	29.17 (8.41)
Male, *n* (%)	23 (95.83)
Home region, *n* (%)	
Central Jutland	13 (54.17)
Zealand	5 (20.83)
Other	5 (20.83)
Single, *n* (%)	11 (45.83)
No children, *n* (%)	18 (75)
Highest completed education, *n* (%)	
Primary or high school	10 (41.67)
Short education (≤ 3 years)	8 (33.33)
Bachelor’s degree (3–4 years)	4 (16.67)
Higher education (≥ 5 years)	2 (8.33)
Gambling debt, mean DKK (Q_1_ – Q_3_^a^)	63,550 (0–55,000)
Type of problematic gambling, *n* (%)	
Slot machines	9 (37.50)
Sports betting	15 (62.50)
Casino	6 (25)
Other^b^	5 (20.83)
Number of GD symptoms (0–10)^c^, mean (SD)	6.96 (1.66)
Other psychiatric disorder, *n* (%)	4 (16.67)

^a^ Lower quartile (25%) to upper quartile (75%). ^b^ Other types of gambling were lotteries, scratch cards, poker, skin betting, and crypto trading. ^c^ According to DSM-V; 4–5 symptoms = mild GD, 6–7 symptoms = moderate GD, 8–9 symptoms = severe GD

### Use of Intervention

Among 17 treatment completers, the mean number of modules completed including the introduction module was 7.35 (SD = 1.06). An overview of data on patient interactions with the treatment platform and therapists can be found in Table 3.

**Table 3:** Patient interaction with treatment platform and therapist

	Completers (*n* = 17)		Non-completers (*n* = 7)	
	Mean (SD)	Range	Mean (SD)	Range
Number of modules completed	7.35 (1.06)	5–8	1.86 (0.90)	1–4
Total length of intervention (weeks)^a^	10.41 (2.83)	8–16	3.57 (1.71)	1–6
Minutes spent on platform	307.38 (144.78)	106–653	84.2 (85.54)	21–220
Number of logins on platform	27.44 (25.30)	7–112	5.4 (2.51)	3–9
*Messages sent to therapist*				
Number of messages	10.38 (5.62)	2–25	2.8 (3.63)	0–9
Characters per message	663.71 (414.56)	119–1619	254.93 (419.26)	0–1001
*Messages received from therapist*				
Number of messages	11.19 (3.08)	6–17	3.6 (2.07)	2–7
Characters per message	716.21 (491.33)	111–1907	494.33 (175.38)	344–771
*Therapist support outside platform*				
Number of supportive sessions^b^	1.94 (1.14)	0–4	0.71 (0.45)	0–1
Total duration of sessions, minutes	108.5 (64.23)	0–230	41.43 (26.42)	0–60
Number of phone calls	1.94 (2.60)	0–5	1.14 (0.99)	0–3
Total duration of calls, minutes	29.94 (57.06)	0–240	17.43 (11.45)	0–30

^a^ From first login on platform until end of treatment or dropout (last contact)

^b^ In-clinic or via online connection

Despite having the opportunity for receiving up to four supportive sessions with their therapists, patients received on average two supportive sessions, spending averagely 54 min per session. As indicated by the large standard deviations and broad ranges in Table 3*,* the amount of therapist support needed varied greatly among patients. A total of 58.3% of logins to the treatment platform happened outside regular clinic opening hours (defined as Monday to Friday from 8 a.m. to 4 p.m.; public holidays excluded). An overview of login times can be found in Figure A (see Supplementary).

### Utility and Acceptability of Intervention

Prior to start of treatment (T2), the mean expectancy score measured with the CEQ was 76.85% (SD = 13.78), indicating the participants having relatively high expectations towards the treatment (100% representing the highest possible expectations). Fifteen participants completed the utility and satisfaction questionnaires post-intervention. Of these, 14 were treatment completers. At the end of treatment, the mean satisfaction score measured with the CEQ was 74.59% (SD = 17.62), corresponding to a relatively high overall satisfaction with the treatment received. The majority of participants reported being highly (*n* = 8; 53.33%) or mostly (*n* = 6; 40%) satisfied with the internet treatment program. Fourteen participants reported that receiving treatment via an internet platform had worked “*well*” (*n* = 4; 26.67%)) or “*really well*” (*n* = 10; 71.14%), and 14 reported having had a sufficient amount of contact with their therapist “*most of the time*” (*n* = 3; 20%) or “*all of the time*” (*n* = 11; 73.33%). An overview of data regarding program utility and participant satisfaction can be found in Figures B, C, and D (see Supplementary).

### Changes in GD and Gambling Behavior

The mean improvement in self-reported GD (measured with NODS) was 4.06 (SD = 2.17) (Table 4). This improvement corresponded to 1.6 standard deviations on baseline NODS scores. The within-group difference in NODS scores from T1 to T3 was found to be significant on a repeated t-test (t (15) = − 7.47, *p* < 0.001), indicating that completers improved significantly on NODS. The NODS cure rate (i.e., the proportion of patients with NODS scores changing from above to below the clinical cut-off from T1 to T3) was 44% (*n* = 8). According to clinician ratings, 12 participants (50%) were in early remission from GD at T3. Eight participants providing data at T3 still reported NODS scores above the clinical cut-off, and by clinician rating all seven non-completers were deemed not in remission and to have benefitted little from treatment. Self-reported gambling behavior decreased significantly from T1 to T3 (Table 4). Interestingly, the change in gambling behavior already occurred at T2 (shortly *before* beginning of treatment), and as such, the decreased gambling behavior at T3 marked a maintained decrease from T2, rather than a decrease *following* treatment (see Discussion).

**Table 4:** Changes in gambling behavior and Gambling Disorder symptoms

	T1 (*n* = 24)	T2 (*n* = 24)	T3 (*n* = 16)	Change		
				T1-T2 (*n* = 24*)*	T1-T3 (*n* = 16)	T2-T3 (*n* = 16*)*
*Gambling behavior past week*						
Number of gambling sessions, mean (SD)	8.42 (22.65)	1.08 (2.47)	0.44 (0.89)	− 7.34*	− 7.98*	− 0.64
Hours spent, mean (SD)	5.67 (13.60)	0.83 (1.06)	0.88 (1.89)	− 4.84*	− 4.79*	+ 0.05
Money spent in DKK, mean (SD)	2,789 (5,015)	664 (2,101)	750 (2,016)	− 2,125*	− 2,039*	+ 86
Gambling problems (NODS)	6 (2.50)	–	1.81 (2.29)	–	− 4.06*	–

^*^ Significant at *p* < .05

*NODS* The National Opinion Research Center DSM Screen for Gambling Problems, modified assesment period: Past month. 1–2 = mild sub-clinical gambling problems, 3–4 = moderate sub-clinical gambling problems, 5–10 = clinical gambling problems, probable GD. T1: Baseline, T2: Post intake-interview, before treatment, T3: Post treatment

### Changes on Secondary Outcomes

**Fig. 3:**
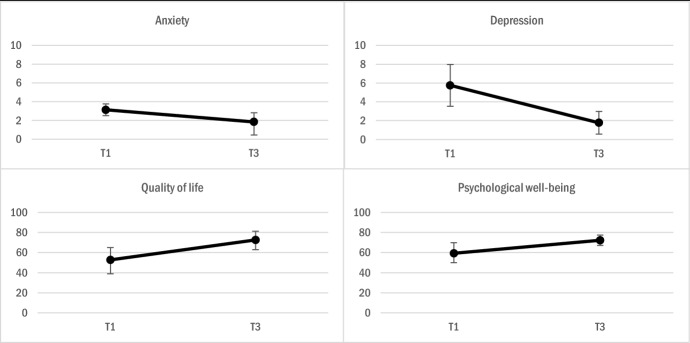
Changes in secondary outcomes. **Anxiety** measured on *SCL-anxiety* [min. score: 0 – max. score: 16]; **Depression** measured on *SCL-depression* [min. score: 0 – max. score: 24]; **Quality of life** measured on one item, where 0 = ”the worst possible life” and 100 = “the best possible life”; **Psychological well-being** measured on *WHO-5* [min. score: 0 – max. score: 100]. All assesment points reported with 95% confidence intervals.

Visual inspection of graphed secondary outcome data indicates a decline in symptoms of anxiety and depression, paired with improvements in psychological well-being and quality of life (Fig. 3).

On the CGI_I, 11 participants were deemed by clinician rating to have benefitted from treatment to *“a high degree”* (*n* = 5, 20.83%) or to *“a very high degree”* (*n* = 6, 25%). By self-report, respectively nine and three participants rated their overall health to be “*better*” or “*much better*” at T3, and all positive change was attributed by participants to either *“the SpilleFri treatment”* (*n* = 3) or to *“both the SpilleFri treatment and other reasons”* (*n* = 8). One participant reported a worsening in overall health at T3 and attributed the worsening to *“reasons outside treatment”.*

### Qualitative Findings: Patient Perspectives

Six participants were recruited for qualitative interviews. Two participants recruited for interviews dropped out of treatment, making the dropout rate of the interview sample about equal to the dropout rate of the overall sample. One dropout participant agreed to participate in an interview after the dropout (interview statements from this patient are denoted “*DO”* in the following).

The thematic analysis of patient interviews and written responses to open-ended questionnaire items yielded themes in three overall categories: 1) Unique benefits associated with *SpilleFri* as compared to face-to-face GD treatment, 2) Practical and psychological barriers, 3) The importance of therapist guidance and alliance. Themes, main findings, and quotes for each overall category are found in Tables 5, 6 and 7.

**Table 5:** Unique benefits associated with *SpilleFri* as compared to face-to-face GD treatment

Theme	Findings	Quotes
Flexibility	Practical benefits associated with flexibility (reduced transport, treatment outside clinic opening hours) means less strain on daily activities	*“I am quite busy in my everyday life. Therefore, I wouldn’t like having to drive to the clinic once a week or so.”* (DO)
		*“The online access makes it very easy and simple. I only have to take one hour out of my weekly schedule.”*
	Psychological benefits associated with flexibility: Possible to engage in treatment when in the right mind-set	*“It is very flexible, and I think that is a good thing. It means that you can work when you feel like you have the motivation. It is up to yourself to find a time when you are in the right mindset.”*
Discretion	Discrete online access to treatment reduces stigma	*“If you have to drive for two hours, it’s a whole day you need to clear for one session. What are you going to tell people? Where did you go to? In that way, SpilleFri is great”* (DO)
*“Being your own therapist”*	*“Being your own therapist”* improves self-discipline	*”You are practicing self-discipline. And that is actually one of the most important things to take home from the treatment, I think. It is also up to your self-discipline to maintain the ‘no’ to gambling. And you learn that through this because it is all up to you alone.”*
Resource bank	Revisiting session content has therapeutic value	*“I shed a tear the second time I entered the module. I mean, I still knew what my goals were, but reading them again… It gave me some peace of mind that things would work out. Despite sitting there having a bit of a hard time imagining going through another week without gambling. It was a bit therapeutic for me.”*
	Possible to share treatment process and progress with next of kin	*"You can actually show off some work to your relatives: Look at this, I'm actually doing something! There is actually something I am working on. I don't just sit and watch videos… Something actually goes on in treatment.”*
Progression made clear and visible	Revisited session content visualizes progression	*"Sometimes I re-read old exercise answers when I need to evaluate myself… Then I can say: OK, well, I'm way beyond THAT point. It gives you an important pat on the back.”*
	The structure of the program makes treatment progression concrete	*"The further you get, the more you can see that there is this common thread. And you can see that you get to the right things in chronological order, right? Module 1, module 2, module 3… You know what you have done and where you are going.”*
Deep and undisturbed reflection – and post-reflection	The act of writing provides special, undisturbed immersion	*”[In conversations] you are more on a stage where you have to defend yourself in front of another person. Whereas here, you actually sit by yourself and have to defend yourself to yourself, and this means having more time to think about your arguments, but also that you… you are more honest with yourself."*
	The written work leads to post-reflection	*“Spending half an hour where you really just sit: How does something make you feel? And having to try to articulate it on a piece of paper… I get so deeply concentrated that I can't really think about anything else. And the flow of thought you had while you sat and did it, well, you get it again when you come across the same problem: That you want to gamble at one point or another."*

**Table 6:** Practical and psychological barriers

Theme	Findings	Quotes
Lack of alliance	With the program: Low confidence in program Lack of individual adaptation	*"If you watch some of the videos and then think: This doesn't fit me, and it’s a little off topic, then you lose the energy to sit and watch it."* (DO)
	With the therapist: Asynchronous contact Too many questions in the message function	*"If I write a message, and then I might get an answer the next day or in two days… At that time, you’re just in other thoughts"* (DO) *"Instead of sending messages when [the therapist] wants to elaborate on a long list of questions, perhaps you could agree to talk on the phone instead? So that you don't have to sit and formulate an entire essay for [the therapist]…"*
	Cold digital contact	*”A message can be a bit cold, that is, you can't see anything but the text. There is just a little more connection with a voice message.”* (DO)
*”Being your own therapist”*	Loneliness and lack of validation Insecurities	*"When you seek help, you need to talk to someone and be told that there is still hope. If you have to deal with [the treatment] yourself, then you still feel that you yourself are the problem…”* (DO) *"You don't feel like you are doing a good enough job… Have you done the exercises properly? Have you actually gained the self-control you think you have? And was it only because I had help sheets? Is this going to go well?”*
When treatment makes demands that exceed resources of the patient	Reading, writing – and patience	*“The modules where there is a lot of text can be a bit demotivating… I can understand what is written, but it is not something I can necessarily relate to and take in."*
	Initiation and organization	*"I'm busy in my everyday-life, so it would have been better to put a meeting in the calendar: Then it would have been more disciplined, and not something I could just reschedule like: OK, I'll watch the video again tomorrow or the day after tomorrow."* (DO)
	Self-discipline and work effort	*"It requires that you to find the energy for a work effort. It's not just about doing all the tasks… You also have to deal with it a lot afterwards, so of course it's a big job you have to do."*

**Table 7:** The importance of therapist guidance and alliance

Theme	Findings	Quotes
Function of therapist	Creates structure and mutual commitment	*"The fact that there is a person sitting ‘on the other side’ means that you are not just sitting with a computer. You have another person who believes in you and has certain expectations of you. You want to fulfill them because you don't want to disappoint another person, like you did when you were a gambling addict."*
	The therapist is *"the warmth in the program":* Validation and digital togetherness	*“Being able to write with a therapist, a real person in there… And the fact that you know who it is, and [the therapist] knows how you feel… For me, that was quite essential.”*
	*Blended care:* Conversations and messages about important life topics not covered by the *SpilleFri* modules	*"In [the conversations with the therapist] I was also able to talk about some more personal things."*
Therapist support can reduce psychological barriers	The therapist's specialist knowledge strengthens confidence in the program and provides an experience of individual adaptation	*"Knowing who is sitting on the other side of the screen: Someone who knows a lot about this condition […] For me, it was crucial in enabling me to trust this computer program actually being able to help me.”*
	The therapist relationship reduces loneliness and insecurities about *"being your own therapist"*	*"The contact with the therapist means that you don't have to deal with it all alone. That's good because otherwise it can seem overwhelming.”*
	The therapists impose structure, reducing demands for initiation and organization	*"For me, it's more disciplined when there's someone watching over me. Like this: Okay, you have to do this module this week.”* (DO)
	Conversations with therapist reduce the workload of reading and written contact	*"You can speak more words than you can read. When we talk for an hour, it corresponds to everything that is written in several modules.”* (DO)

### Qualitative Findings: Acceptability Among Therapists

Overall, the therapists were positive towards the potentials of the *SpilleFri* treatment, but also experienced certain limitations. Importantly, the therapists found the manualized intervention to be insufficient for patients experiencing significant ambivalence regarding the decision to stop gambling. To meet the needs of patients with high degrees of ambivalence, the program would need more motivational content or, alternatively, ambivalent patients would need a number of face-to-face sessions focused on motivational work before starting treatment in *SpilleFri*. In general, the therapists believed the supportive sessions to be important in retaining patients in treatment – especially in cases of ambivalence, or when patients presented with individual problems alongside the gambling problems demanding individually tailored treatment content.

## Discussion

### Principal Findings

The main finding of this pilot feasibility study was that the therapist-assisted internet-based program *SpilleFri* was a feasible treatment for patients with GD. The predefined feasibility criteria for the dropout rate and data completeness were met: seven (29.17%) patients dropped out, corresponding to a low dropout rate compared to previous studies of guided iCBT for GD (Carlbring et al., 2012; Melville et al., 2007). Furthermore, 82.35% of treatment completers and 66.67% of all included participants provided full data on primary outcome measures at the end of treatment.

Logged data from the treatment platform showed that patients made frequent use of the possibility to attend treatment outside regular clinic opening hours, and in general, the qualitative sub-study showed that, the flexibility of the online format helped patients overcome a number of practical barriers typically associated with attending face-to-face treatment for GD. Similarly, the qualitative analysis yielded insights into how psychological treatment barriers associated with stigma and desire to self-manage problems could be mitigated by the distinctive therapeutic process associated with internet-based treatment. Therapists delivering the intervention also found the treatment program to be acceptable, albeit maybe better suited for patients with milder GD severity and less ambivalence.

Like the iCBT studies included in the meta-analysis by Sagoe and colleagues (2021), we found indications of positive effects on GD severity, gambling behavior, and related areas of functioning such as quality of life, well-being, anxiety, and depression in treatment completers. Taken together, the present feasibility study found the study procedures and the treatment program to be feasible and acceptable. The promising outcome results also suggest potential efficacy of the *SpilleFri* treatment program, encouraging a prospective effect study.

### Perspectives for Further Increasing Treatment Availability and Reducing Barriers

Despite mitigated treatment barriers, such as reduced stigma, reduced strain on daily life, and increased experiences of autonomy in treatment, our recruitment of patients through the usual treatment recruitment channels may have limited the reach of the internet treatment platform. It is possible that recruitment through other channels (e.g., social media, a gambling help-line, or directly via online gambling sites) would extend the reach. In the context of a Swedish internet-based GD intervention recruiting patients in the context of a gambling helpline proved “relatively easy” (Wall et al., 2021) – this study, however, found very high attrition rates (90% after 14 days).

As patients appreciated the anonymity and flexibility of the *SpilleFri* treatment, extending the anonymity and flexibility of the recruitment process (e.g., by conducting intake interviews via telephone or online) could potentially maximize the barrier mitigating effects of the online format. Possibly, an entirely internet-based recruitment process could make the treatment platform more attractive for non-treatment seeking individuals. Recruitment barriers related to problem denial, however, are generally very hard to overcome (Gehlenborg et al., 2021).

Systematic involvement of concerned significant others (CSOs) in treatment might decrease dropout from internet-based GD treatment. For example, Nilsson and colleagues (2020) found that involving CSOs in internet-based GD treatment slightly increased treatment adherence. The therapists participating in our focus group interviews found involving CSOs to be very important for successful treatment processes and requested for CSO involvement to become a formal part of the *SpilleFri* platform. Potentially, a supplementary treatment module encouraging gamblers to involve CSOs or a mirror-platform aimed directly at CSOs could limit treatment dropout and improve overall treatment outcome in the future.

### Strengths

First, an important strength of this feasibility study was the thorough diagnostical assessment at patient intake. As shown in the meta-analysis by Sagoe and colleagues (2021), lenient inclusion criteria based only on self-reported screening questionnaires are often utilized in studies of internet-based GD interventions, resulting in less well-defined participant groups. To our knowledge, the current study is the first study offering iCBT to clinically diagnosed GD patients.

Second, the evaluation of treatment feasibility was strengthened by the inclusion of data across multiple data sources, including self-report, logged activity on the treatment platform, clinic records, and clinician-rated outcomes.

Third, our combination of quantitative and qualitative data collection allowed for a broader, more in-depth, and more contextualized evaluation of treatment feasibility and acceptability as experienced by stakeholders (patients and therapists) than would have been possible with a purely quantitative approach. Our mixed-methods approach follows the recommendations of the framework for developing and evaluating complex interventions, developed by the Medical Research Council (Skivington et al., 2021).

Lastly, unlike most similar studies, we assessed the primary outcome variable before and after the intake interview as well as after treatment. Importantly, the addition of an assessment point after the intake interview and immediately before start of treatment revealed that the main improvement in patient gambling behaviour occurred *before* patients began treatment, and thus, the observed significant change in self-reported gambling behaviour from baseline to post-treatment might be interpreted as a maintained change during treatment rather than a change caused directly by the treatment program.

### Limitations

First, no control group was used, and so, no conclusions can be drawn regarding the efficacy of the intervention. The present findings indicate, however, that both the treatment platform and the study procedures are feasible for conducting a randomized controlled trial with a larger sample size.

Second, as no follow-up assessment was made, we do not yet know if the observed changes in gambling behavior and symptom severity persist.

Third, as most patients who left the *SpilleFri* treatment before completing six modules did not respond to post-treatment questionnaires, they could not be included in the feasibility analysis, and we generally know little of their reasons for leaving the treatment early; with the exception of a single patient who agreed to participate in an interview after dropout. For the same reason, unknown adverse consequences of treatment cannot be ruled out.

Lastly, as our sample was relatively homogenous and included only one female patient, the observed positive outcomes cannot be generalized to more heterogeneous samples including patients with moderate to severe comorbidity, more female patients, and patients over the age of 35.

## Conclusion

Implementation of internet-based treatment opportunities has been proposed as a way of mitigating practical and psychological barriers that may deter patients with gambling disorder from seeking and receiving treatment. Here, we showed that a guided internet-based cognitive behavioral treatment program may be a feasible treatment set-up for patients suffering from GD, and that such internet-based treatment platforms might indeed help patients overcome the practical and psychological barriers typically associated with face-to-face treatment for GD. Our qualitative analysis suggested that not only did the *SpilleFri* treatment approximate the processes of traditional treatment, but in several areas the online format seemed to elicit qualitatively different treatment experiences. The observation that the included patients generally experienced positive effects of being made responsible for their own recovery possibly indicates a good match between the patient group's need for autonomy and the patient independence inherent to internet-based treatment, underlining the potential for further development and implementation of internet-based treatment specifically for patients with GD.

The uncontrolled design and small sample size of the study limited the robustness of the findings. Therefore, the findings should be replicated in a randomized controlled trial. Potentially, *SpilleFri* may contribute to increase the availability and accessibility of specialized treatment for GD.

### Supplementary Information

Below is the link to the electronic supplementary material.

Supplementary file1 (DOCX 161 KB)

## Data Availability

The datasets generated and analyzed during the current study are not publicly available due to the privacy of the included patients but are available from the corresponding author on reasonable request.
