# Supplementary material for: “I was Treated by the Program, the Therapist, and Myself”: Feasibility of an Internet-Based Treatment Program for Gambling Disorder
Source: J Gambl Stud. 2023 Mar 31;39(4):1885–907. doi: 10.1007/s10899-023-10199-x (PMC10628035; doi:10.1007/s10899-023-10199-x)
Supplement: Supplementary file 1 — Supplementary file1 (DOCX 161 KB) [file 10899_2023_10199_MOESM1_ESM.docx]

# Supplementary material

**Figure A. Login time and day**

| Total number of logins | 100 |
| --- | --- |
|  | 80 |
|  | 60 |
|  | 40 |
|  | 20 |
|  | 0 |


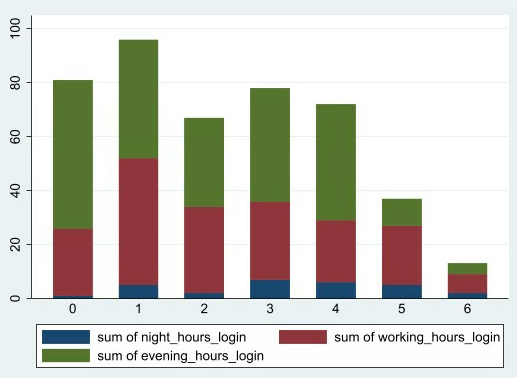

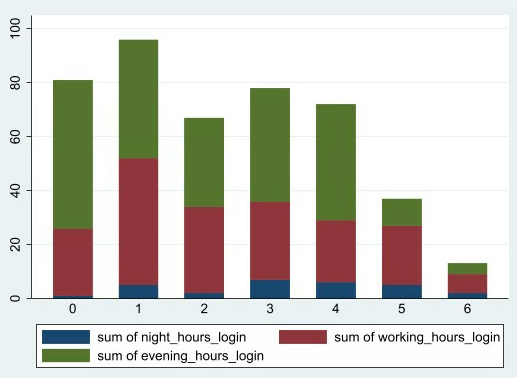


*Thursday*

*Sunday*

*Saturday*

*Friday*

*Wednesday*

*Tuesday*

*Monday*

Night hours (00.00-07.59)

Evening hours (16.00-23.59)

Working hours (08.00-15.59)

**Figure B. Satisfaction with the internet program**

**3.47**

**3.27**

**3.33**

Extremely

Mostly

Somewhat

A little

Not at all

**2.93**

*n* = 15

Measured on a 5point Likert-scale from 0 (‘*not at all’*) to 4 (‘*extremely’*)

**Figure C. User-friendliness of the internet program**

*n* = 15

**Figure D. Participant opinion on treatment content**

*Psychoeducational videos with representative from the gambling industry

**Via telephone or face-to-face
